# Measurement of overgeneral autobiographical memory: Psychometric properties of the autobiographical memory test in young and older populations

**DOI:** 10.1371/journal.pone.0196073

**Published:** 2018-04-19

**Authors:** Laura Ros, Dulce Romero, Jorge J. Ricarte, Juan P. Serrano, Marta Nieto, Jose M. Latorre

**Affiliations:** Department of Psychology, University of Castilla La Mancha, Albacete, Spain; University of St Andrews, UNITED KINGDOM

## Abstract

The Autobiographical Memory Test (AMT) is the most widely used measure of overgeneral autobiographical memory (OGM). The AMT appears to have good psychometric properties, but more research is needed on the influence and applicability of individual cue words in different languages and populations. To date, no studies have evaluated its usefulness as a measure of OMG in Spanish or older populations. This work aims to analyze the applicability of the AMT in young and older Spanish samples. We administered a Spanish version of the AMT to samples of young (*N* = 520) and older adults (*N* = 155). We conducted confirmatory factor analysis (CFA), item response theory-based analysis (IRT) and differential item functioning (DIF). Results confirm the one-factor structure for the AMT. IRT analysis suggests that both groups find the AMT easy given that they generally perform well, and that it is more precise in individuals who score low on memory specificity. DIF analysis finds three items differ in their functioning depending on age group. This differential functioning of these items affects the overall AMT scores and, thus, they should be excluded from the AMT in studies comparing young and older samples. We discuss the possible implications of the samples and cue words used.

## Introduction

Autobiographical memory (AM) may be defined as a mental representation of the events of one’s past, containing episodic memories and self-referential semantic information [1]. Overgeneral autobiographical memory (OGM) is one of the most studied aspects of AM. It may be defined as the difficulty in retrieving specific memories (periods of time measured in seconds, minutes and hours that last less than 24 hours, for example, “my wedding day”) and recalling general memories instead. Within the general memories, two types of memories are distinguished: categoric memories (memories of events stored in categories such as persons, places and activities, for example, ‘every time I went to the beach’) and extended memories (memories that refer to an event lasting for more than one day, for example, “when I was at school”) [2]. In recent years, there has been an increase in research relating OGM to psychopathologies such as affective disorders, post-traumatic stress disorder, acute stress, schizophrenia or personality disorders [3–6].

According to Conway and Pleydell-Pearce [7], specific memories can be retrieved by two processes: generative retrieval and direct retrieval. Generative retrieval refers to top-down search processes involving the use of conceptual representations. During this process, mnemonic cues and criteria are established. These cues and criteria will form the basis for memory search and activate the general event level knowledge needed to tap into specific memories. Direct retrieval, by contrast, corresponds to the subjective experience of spontaneous recall, and emerges when an internal or environmental cue produces an immediate activation of specific memory. This type of memory is more rapid and less resource demanding than generative retrieval. According to Williams [8], overgeneral memory emerges when individuals truncate their search during generative retrieval at too high a level, when only general information has been accessed, rather than moving down to specific event memory level. Williams [2] developed the CaRFAX model to explain the causes for the occurrence of OGM. This model comprises three different mechanisms, which, either alone or in combination, underlie deficits in specificity: capture and rumination (CaR); functional avoidance (FA); and impaired executive control (X). Capture and rumination occur when conceptual self-relevant information activates rumination processes during retrieval, “capturing” cognitive resources and disrupting the retrieval search. Functional avoidance refers to the avoidance of the retrieval of specific memories as a means of negative affect regulation. It occurs when the memory search is aborted at a general event level as a result of a passive avoidance reaction. The last mechanism, impaired executive control, refers to deficits in executive functions that limit the successful retrieval of specific memories. Generative retrieval requires use of executive resources to set up a retrieval model and to compare it with retrieved information, and to inhibit information that is irrelevant to the memory being sought [7].

A number of very different methods have been used to study AM [1], but most research on OGM has used the Autobiographical Memory Test (AMT), based on methodology originally developed by Williams and Broadbent [9]. In the AMT, participants are presented with a series of cue words, for which they are asked to produce a specific memory. The memories provided are then coded according to level of specificity. However, different versions of the AMT have been employed across studies. Studies have varied in the emotional valence (positive, negative and/or neutral) and the number of cue words used. This number tends to be between 10 and 20 cue words. Regarding the emotional valence, studies usually use positive and negative cue words, while neutral words are less frequent [1,10]. They also differ in whether prompting is provided when participants fail to recall a specific memory. In some studies, prompting is used to determine whether the participant can generate a specific response if his or her first response is non-specific. For example, if the participant’s first response is not a specific memory, additional queries are used to facilitate the retrieval of a specific memory (e.g., “but you can describe a specific day when you felt…?). The use of prompting evidently increases the retrieval of specific memories [8,10]. Finally, studies vary in the amount of time participants are given to respond to a particular cue. According to the review by Griffith et al. [1], most studies use a time limit of either 30 or 60 seconds, while in some studies, however, there is no time limit and the participant sets his or her own response pace. AMT procedures with shorter response time limits may be viewed as more demanding because individuals are required to generate a specific memory more quickly [11]. So, individuals who experience greater difficulty retrieving specific memories would be likely to perform worse on tests with shorter response time limits [10].

Various studies underline the need to further analyze the psychometric properties of the AMT in measuring OGM and also suggest that data analysis should be conducted using item response theory (IRT) models instead of traditional statistical models, which use the sum of the different items [1,12]. IRT is an item-oriented, rather than a test-oriented, approach to psychometric analysis. IRT analysis of the AMT can provide information on how difficult it is to generate a specific memory in response to a particular cue word and how well a cue word discriminates between different levels of memory specificity ability. In this sense, IRT analysis might provide information to help improve word selection. Additionally, IRT analysis also informs about the test accuracy providing an estimate of the measurement error of the test. Accuracy refers to the precision with which an individual’s level of ability on the test can be estimated. The precision/accuracy increases when the measurement error decreases. Thus, the highest precision of the test is reached at the point where their measurement error is lowest.

Several studies have analyzed the psychometric properties of the AMT. The first was conducted by Griffith et al. [13] with three separate samples of healthy young adults (aged 16 to 36). Their results support the existence of a single trait of AM specificity, comprising the combination of positive and negative cue words. This one-factor structure is also confirmed in the studies conducted by Heron et al. [14] in an adolescent population, those by Nuttall et al. [15] and Nieto et al. [16] with preschool-age children, as well as the studies by Griffith et al. [12] in a sample of recent trauma survivors and by Takano et al. [17] with a Japanese community sample.

Regarding the accuracy of the AMT in measuring OGM, Griffith et al. [12] find that the AMT is more accurate in individuals with low memory specificity scores. In other words, the test is more precise in individuals who score low on memory specificity compared to other participants. By contrast, studies in adolescent population [14] and preschool population [16] find that the test is more accurate in individuals with medium memory specificity scores. Griffith et al. [13] state that, in a young population, the AMT is an easy task as their results show that the cue words are more likely to generate a specific memory even in individuals scoring below the mean on memory specificity.

It must be noted that studies using AMT have mainly been conducted in Western societies. Few studies have investigated autobiographical memory specificity in Eastern populations [17–20]. Results show that Eastern populations tend to recall autobiographical memories with fewer specific details than Western populations [17,21]. These results are argued to be rooted in the differences in educational styles between the two cultures: in Eastern populations, social roles are emphasized and, as a result, social harmony is of great importance; in Western populations, however, personal autonomy is emphasized, so expressing emotions is encouraged [22,23]. Nonetheless, other studies find no differences in autobiographical recall between these cultures [24,25]. Takano et al. [17] found lower levels of AM specificity in a Japanese sample (age range: 16–81 years) compared to Belgian and UK samples. Nevertheless, they found that the youngest age Japanese group had comparable levels of AM specificity (around 40%) to the previous data from European student and child samples [14,26] and that the levels of AM specificity had a significant declining trend as a function of age. For these reasons, they suggested that this difference in AM specificity measured by the AMT was not attributable to cultural differences, but mainly to age differences between both samples (Japanese vs Belgian and UK samples). Finally, their study replicated the one-factor structure of the AMT and they concluded that the AMT had robust psychometric properties across different languages and cultural backgrounds.

Despite the existing body of research on AM, Griffith et al. [1] propose that additional work is needed to better elucidate how procedural differences affect the measurement of OGM. These authors point to the importance of determining the characteristics of cue words that influence AMT performance, as this would inform the selection of cue words for studies with different populations and, to the extent possible, would enable the production of standardized word lists with known properties for precise measurement of OGM, increasing the comparability of the results across investigations. However, they also suggest there might be circumstances in which cross-study standardization would be undesirable (e.g., if different cue words were found to be differentially related to OGM in different populations). To the best of our knowledge, there are currently no studies validating the AMT in Spanish (except that by Nieto et al. [16], in a preschool population). It is important to conduct this type of validations in different languages/cultures in order to establish a standardized set of cue words adapted to different populations.

OGM has been associated with older populations. Several studies suggest that, compared to younger adults, older adults exhibit greater OGM. Following the CaRFAX model [2], the greater OGM in older adults could be a result of age-related decline in executive function [27,28]. In fact, this association between low executive capacity and greater OGM has also been found in an adult population [29,30]. In the same vein, a study by Griffith et al. [31] in a sample of adults diagnosed with major depressive disorder found that age is a better predictor of OGM than variables such as a diagnosis of PTSD or number of depressive episodes. Takano et al. [17] also found that AM specificity declined significantly as a function of age. Thus, age can be considered an important predictor of OGM. If older adults exhibit higher levels of OGM than young individuals, could there be age-related differences in AMT performance? As stated by Barnhofer et al. [32], the level of cognitive function impacts on AMT performance. In their study, they found that participants showed greater OGM when presented with self-relevant cue words on the AMT under conditions of high–but not low- cognitive load. These results would support the idea of an interaction between impaired executive control, and capture and rumination. Additionally, Griffith et al. suggest that the AMT may be too easy for individuals with a high level of cognitive function [13]. In their study, they compared two versions of the AMT: traditional instructions (giving a specific memory for each cue word) vs. minimal instructions (generating an autobiographical memory for each cue word, without explicitly stating that the memories should be specific). Their results showed that the thresholds for specific memories were higher for the minimal instructions group compared to the traditional instructions group where the thresholds were too low. These results suggest that the traditional instructions AMT could result easy to participants with a normal cognitive functioning. For this reason, Griffith et al. concluded that the minimal instructions version may be more useful in samples with an average memory functioning (e.g., college samples), whereas the traditional instructions version may be useful in samples with poor memory functioning (e.g., clinical samples). Normal aging is often accompanied by a deterioration of different cognitive skills, so might there be age-related differences in how the AMT cue words function?

Reliability and validity are two characteristics that all measurement instruments must have, including psychological tests. Any test parameter, such as item difficulty or discrimination, that is different between two or more subpopulation groups, may be a sign of a threat to test validity because the test results would need different interpretations for each group. In this context, differential item functioning (DIF) is an important validity and bias issue of test analysis [33]. To establish whether older people produce fewer specific memories in the AMT because they are more overgeneral than younger participants, we should previously verify that the AMT presents no DIF between the groups, because the presence of DIF can lead to biased results when examining differences between groups. To our knowledge, there are still no studies analyzing the psychometric functioning of the AMT in samples of older adults nor whether the cues words differ in their function according to age.

This study has two main objectives. The first is to validate a Spanish version of the AMT for adult populations (young and older samples). To this end, we aim to confirm the one-factor structure of the AMT and then, using an IRT analysis, we examine the individual characteristics of the AMT items and evaluate the precision of the AMT in measuring OGM in young and older participants. The second objective is to analyze the DIF of the AMT cue words in young and older samples. Consequently, our aim is to determine whether any of the cue words are differentially related to OGM according to age group (young vs older).

## Method

### Participants

One hundred and fifty-five older adults participated in this study (97 men and 58 women; ages 55–88 years; *M* = 66.86, *SD* = 6.92; educational level: 29.0% completed elementary school, 36.8% completed secondary school, and 34.2% attended university), and 520 younger adults (255 men and 265 women; ages 17–30 years; *M* = 20.20, *SD* = 2.20; educational level: 4.2% completed secondary school and 95.8% attended university). All participants were previously assessed using the Mini International Neuropsychiatric Interview (MINI) [34] to guarantee the absence of psychiatric disorders in the samples at the time of assessment. The group of older adults was formed by volunteers from a cultural association for older people in Albacete and Talavera, Spain (“Asociación de Alumnos de la Universidad de la Experiencia” [ALUEX]). The educational level of members of this association is usually high and they routinely perform intellectual activities. None of them had cognitive impairment, as measured by the Spanish version of the Mini-Mental State Examination (“Mini-Examen Cognoscitivo” [MEC]) [35]. The group of younger adults comprised university students or volunteers from research groups (scholarship holders, research technicians, etc.) at the Faculties of Medicine and Computer Science of the University of Castilla-La Mancha in Albacete (Spain).

### Materials and procedure

To recruit older participants, informative sessions were held in cultural association centers in Albacete and Talavera. Attendees were informed that the Department of Psychology of the University of Castilla-La Mancha were planning to undertake a study on autobiographical memory functioning. Those interested in participating signed informed consent forms and their personal details were noted down so we could arrange subsequent individual data collection sessions. The procedure was identical for the recruitment of young participants except that the informative sessions were conducted by professors from both faculties during classes.

Following the AMT instruction manual [36], the cue words of this version of the AMT were selected from the study by Brittlebank et al. [37] and translated into Spanish (6 positive words and 6 negative words). The translated words were evaluated using a standardized lexical program [38] to ensure there were no differences in frequency, imageability, and familiarity between positive and negative cue words (see Table 1). The final version consisted of ten words: five positive cue words (*happiness*, *friendship*, *excitement*, *energy* and *smile*) and five negative cue words (*guilty*, *failure*, *worry*, *sadness* and *illness*). There were no statistically significant differences in frequency, familiarity and imageability between positive and negative cue words (all *p*s > .175)

**Table 1:** Frequency, familiarity and imageability ratings for the 10 AMT cue words according to Buscapalabras lexical program [38].

Item	Frequency^a^	Familiarity^b^	Imageability^b^
Happiness	46.61	6.04	4.05
Friendship	39.29	6.36	4.08
Excitement	38.21	6.64	4.10
Energy	111.61	5.85	2.75
Smile	98.75	6.18	6.21
Guilty	23.39	5.36	4.33
Failure	43.39	5.50	3.45
Worry	31.79	6.31	3.80
Sadness	36.43	6.82	4.99
Illness	111.25	6.45	5.01

Note.

^a^ Frequency per million words.

^b^ Scores are on a scale from 1 to 7, where higher scores indicate greater familiarity/imageability.

The AMT procedure was taken from Williams et al. [39]. The cue words were presented orally in a fixed order, alternating between positive and negative cue words. Participants were asked to generate a specific memory that happened on a particular day at least one week ago. Each memory recalled had to be different. Prior to the test, the concept of specific memory was explained by use of examples. They were also given two practice words (*car* and *tree*) in order to ensure understanding and clarify any doubts. Participants received the following instruction after each cue word: "Try to remember a day or situation in the past when you felt [*cue word*]. Can you describe it?”. They were given a minute to answer. When the 60-second time limit for a cue was reached, the next cue word was presented to the participants.

Each memory was coded according to its level of specificity. Memories referring to periods measured in seconds, minutes and hours and lasting less than 24 hours were coded as specific memories (e.g., “my wedding day”). Memories referring to an extended period were coded as extended memories (e.g., “when I was at school”). Memories referring to a whole class of events generally stored in categories such as persons, places, or activities were coded as categoric memories (e.g. “every argument with my husband”). Memories derived from general semantic knowledge rather than a memory (e.g. “my friend Maria”) were coded as semantic associates. Finally, the no memory category included memories referring to an event already mentioned in a previous cue word, memories referring to an event from the past week, and no responses to the cue word. Two separate examiners coded all the participants’ memories. Cohen’s κ was run to determine whether there was agreement between the examiners’ coding of the memories. The agreement was strong (κ = .89, *p* < .001).

All procedures followed were in accordance with the ethical standards of the responsible committee on human experimentation (institutional and national) and with the Helsinki Declaration of 1975, as revised in 2000. This study was approved by the Clinical Research Ethics Committee of the Albacete University Hospital. All participants gave written informed consent to participate in the study.

### Data analysis

First, a descriptive analysis of the data was conducted using SPSS 20.0.

Second, a confirmatory factor analysis (CFA) was conducted to test whether the score obtained on the AMT represents a single dimension of the specificity of AM. In addition, we controlled for the possibility of a two-dimensional fit of the data depending on the valence of the cue word (positive vs negative). The psychometric properties of the AMT were examined using IRT. These analyzes were conducted using Mplus 6.12 software [40]. IRT and CFA analyses were conducted independently for older and younger participants.

Finally, we also examined age-group-based differential item functioning (DIF). DIF analysis involves matching members of the reference group (majority or advantaged group; in this case, the young group) and the focal group (minority or disadvantaged group; in this case, older group) on a measure ability (AM specificity) and implementing statistical procedures to identify group differences on test items. The lordif package [41] in R [42] was used to evaluate DIF. DIF tests commonly distinguish between uniform and non-uniform DIF [43]. Swaminathan and Rogers [44] proposed the use of logistic regression in DIF detection for dichotomous items (in this case, specific vs non-specific AMs). Each cue word is considered as the dependent variable in logistic regression models and the independent variables are participants’ ability on the AMT (the trait measured by the test), age group and an interaction term between ability on the AMT and age group. Assessment of uniform and non-uniform DIF is based on comparing three different models: A) Model 1: logistic regression model with ability on the AMT as an independent variable; B) Model 2: logistic regression model with ability on the AMT and age group as independent variables; and C) Model 3: logistic regression model with ability on the AMT, age group and the interaction term between ability and age group as independent variables. Testing for the presence of DIF (both uniform and non-uniform) under the logistic regression framework is traditionally based on the likelihood *χ*^*2*^ test [44]. Uniform DIF indicates changes in item difficulty across groups and is tested by comparing the log likelihood values for Models 1 and 2. Thus, in our study, uniform DIF effects would consist of age differences in item thresholds, but discrimination/factor loadings would not vary across groups. Non-uniform DIF indicates that the relationship between the trait and the item varies across groups, that is, discrimination must differ between groups. Non-uniform DIF is tested comparing the log likelihood values for Models 2 and 3. Lordif also provides estimates of a “total DIF effect” for each item. Total DIF effect is an important measure to take into account because although items might contain DIF (uniform or non-uniform) for certain groups, this does not mean the DIF affects the overall scale scores [45]. This total DIF effect is tested by comparing Models 1 and 3.

To determine the magnitude of DIF, we used McFadden’s *R*^2^ Δ as a measure of effect size. This statistic is defined as the difference in MacFadden’s *R*^*2*^ between the compared models. Jodoin and Gierl [46] recommended the following effect sizes: *R*^2^ < .035, a negligible effect size; .035 ≤ *R*^2^ < .070, a medium effect size, and *R*^2^ ≥ .070 a large effect size.

## Results

### Descriptive statistics

Table 2 show the distribution of responses and the percentages for each of the AMT items. Most participants, both young and old, provided specific memories in response to each AMT item. However, if we consider the total number of specific memories in AMT, we find that the younger group (*M* = 7.93, *SD* = 2.18) provides more specific memories than the older group (*M* = 6.65, *SD* = 2.31; *t*(673) = 6.34, *p* < .001, Cohen’s *d* = 0.57).

**Table 2:** Distribution of responses for the 10 AMT items for younger (n = 520) and older (n = 204) samples.

	Specific		Categoric		Extended		Semantic Associate		No memory	
Item	Younger	Older	Younger	Older	Younger	Older	Younger	Older	Younger	Older
Happiness	454 (87.3%)	188 (92.2%)	27 (5.2%)	5 (2.5%)	8 (1.5%)	10 (4.9%)	10 (1.9%)	-	21 (4.1%)	1 (0.4%)
Friendship	388 (74.6%)	106 (52.0%)	36 (6.9%)	26 (12.7%)	44 (8.5%)	54 (26.5%)	12 (2.3%)	12 (5.9%)	40 (7.7%)	6 (2.9%)
Excitement	416 (80.0%)	147 (72.1%)	40 (7.7%)	21 (10.3%)	28 (5.4%)	17 (8.3%)	6 (1.1%)	8 (3.9%)	30 (5.8%)	11 (5.4%)
Energy	385 (74.0%)	90 (44.1%)	62 (11.9%)	29 (14.2%)	19 (3.7%)	51 (25.0%)	13 (2.5%)	14 (6.9%)	41 (7.9%)	20 (9.8%)
Smile	405 (77.9%)	123 (60.3%)	63 (12.1%)	38 (18.6%)	9 (1.7%)	28 (13.7%)	17 (3.3%)	12 (5.9%)	26 (5.0%)	3 (1.5%)
Guilty	449 (86.3%)	116 (56.8%)	22 (4.2%)	28 (13.7%)	17 (3.3%)	30 (14.7%)	6 (1.2%)	5 (2.5%)	26 (5.0%)	25 (12.3%)
Failure	397 (76.3%)	117 (57.4%)	37 (7.1%)	21 (10.3%)	47 (9.1%)	27 (13.2%)	11 (2.1%)	7 (3.4%)	28 (5.4%)	32 (15.7%)
Worry	386 (74.0%)	125 (61.3%)	62 (11.9%)	18 (8.8%)	19 (3.7%)	48 (23.5%)	13 (2.5%)	9 (4.4%)	41 (7.9%)	4 (2.0%)
Sadness	446 (85.8%)	157 (77.0%)	36 (6.9%)	17 (8.3%)	15 (2.9%)	20 (9.8%)	3 (0.6%)	3 (1.5%)	20 (3.8%)	7 (3.4%)
Illness	396 (76.2%)	127 (62.3%)	17 (3.3%)	16 (7.8%)	66 (12.7%)	41 (20.1%)	18 (3.5%)	7 (3.4%)	23 (4.3%)	13 (6.4%)

### Confirmatory factor analysis

In this analysis, the variance of each factor was set at 1.0 so that the loading of each AMT item could be freely estimated. Items were treated as ordinal categorical indicators and so a weighted least squares means and variance adjusted estimation (VLSMV) was used. In order to evaluate the model fit, the *comparative fit index* (*CFI*) and a *root-mean-square error of approximation* (*RMSEA*) were used. For well-fitting models, cut-off points for *CFI* of ≥ 0.90 [47] and for *RMSEA* of < 0.06 have been suggested, as well as models with non-significant *χ*^*2*^ statistics [48].

As regards the younger group, the one-factor model provided an excellent fit to the observed data as indicated by CFI = .98 and RMSEA = .03. Nevertheless, the *χ*^*2*^(35, *N* = 520) = 55.46 reached statistical significance (*p* = .015). As the significance of the *χ*^*2*^ statistic is influenced by the sample size, some authors suggest the use of *χ*^*2*^/df ratio as a better measure of the goodness-of-fit of the overall model [49]. In this study the ratio is 1.58, lower than recommended by conventional standards (i.e., <2) [50].

As regards the older group, the one-factor model also provided an excellent fit as indicated by CFI = .94, RMSEA = .05, and a *χ*^*2*^/df ratio equal to 1.46 (*χ*^*2*^(35, *N* = 204) = 50.93, *p* = .040).

The two-factor model could not be identified in either group (young and older) as the estimated correlations between the latent variables of the two-factor model exceeded 1.0.

### Item response theory analyses

The IRT parameters were calculated using Samejima’s graded response model [51]. Each of the observed items was related to a latent trait of AM specificity using probit regression analysis. This type of analysis provides two types of parameters: item-slopes (or discrimination parameters) and thresholds (or difficulty parameters). The first parameter provides a measure of the item’s ability to discriminate between low and high scorers in the latent trait studied. In this study, a cutoff of .30 is used as a threshold to identify substantive standardized loadings/slopes [12]. The second parameter provides information on an individual’s level of difficulty in generating a certain response to an item. The two indices together (item-slope and thresholds) determine the probability of giving a specific response to an item depending on the level of ability of a person in the latent trait studied. Additionally, information about Standard Error of Measurement (SEM) is also provided. SEM is a common metric of test imprecision and can be used to construct confidence intervals around individual test scores.

Item slopes and threshold parameters estimates are shown in Table 3 for both groups. In this study, there are five response categories, so our results present four thresholds. The results show standardized item-slopes of .38 or higher (except for *Happiness*, which is below .30) and standardized thresholds ranging from -2.58 to 0.15. In general, the AMT seems to be easy for both samples, since the probability of participants responding with a specific memory is high.

**Table 3:** Item response theory parameters for younger (n = 520) and older (n = 155) samples.

	Item-slope		Threshold 1 (no mem/associate)		Threshold 2 (associate/categoric)		Threshold 3 (categoric/extended)		Threshold 4 (extended/specific)	
Word	Young	Older	Young	Older	Young	Older	Young	Older	Young	Older
Happiness	.76	.29	-1.75	-	-1.56	-2.58^a^	-1.22	-1.89	-1.14	-1.42
Friendship	.67	.64	-1.43	-1.89	-1.28	-1.35	-0.96	-0.79	-0.66	-0.05
Excitement	.61	.64	-1.57	-1.61	-1.48	-1.32	-1.05	-0.86	-0.84	-0.59
Energy	.64	.40	-1.41	-1.29	-1.26	-0.97	-0.76	-0.50	-0.64	0.15
Smile	.58	.53	-1.65	-2.18	-1.39	-1.45	-0.83	-0.64	-0.77	-0.26
Guilty	.44	.38	-1.65	-1.16	-1.54	-1.05	-1.26	-0.57	-1.10	-0.17
Failure	.60	.42	-1.61	-1.01	-1.44	-0.87	-1.05	-0.54	-0.72	-0.19
Worry	.57	.42	-1.68	-2.06	-1.48	-1.52	-1.09	-1.03	-0.65	-0.29
Sadness	.69	.40	-1.77	-1.82	-1.70	-1.65	-1.21	-1.12	-1.07	-0.74
Illness	.63	.63	-1.70	-1.52	-1.41	-1.29	-1.22	-0.93	-0.71	-0.31

Note. All item slopes are statistically significantly different from zero (*p* < .001).

^a^ This item did not generate semantic associates in older group. Consequently, the value shown in the table refers to the threshold from a no memory to a categoric one.

**Fig 1:**
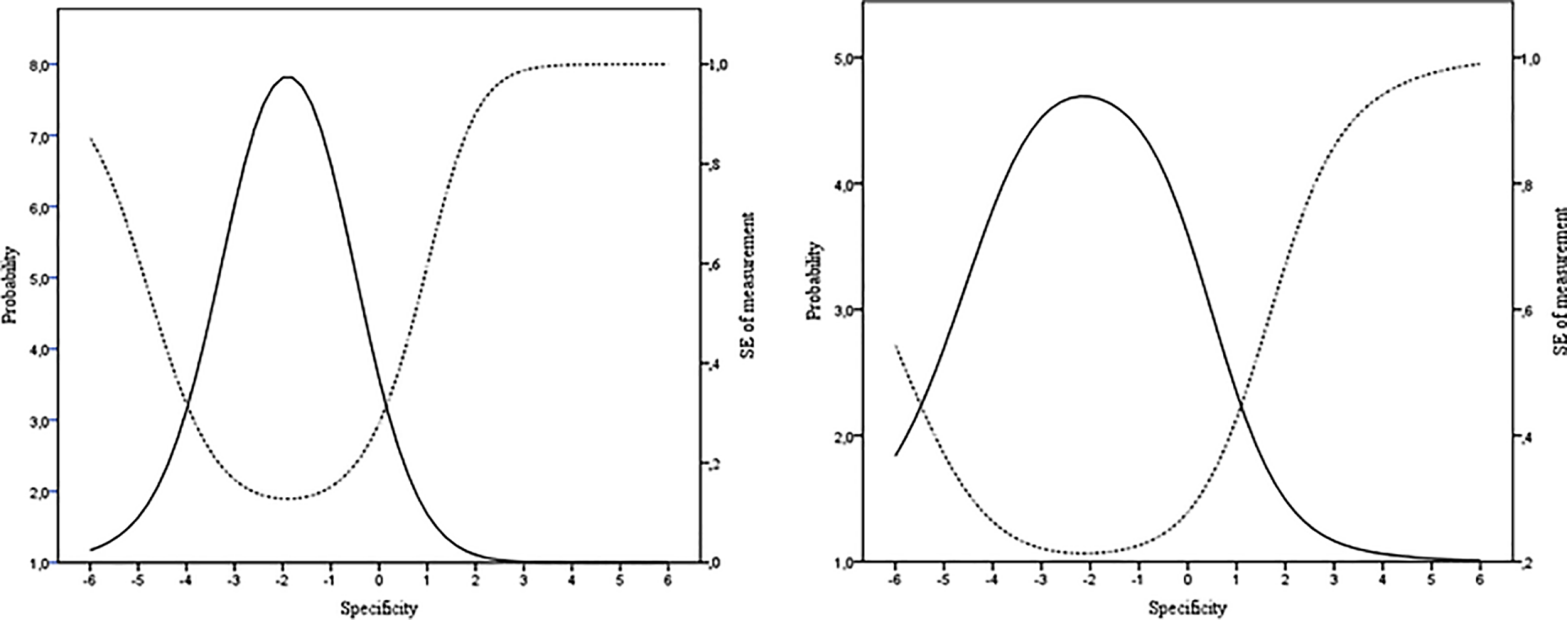
Test information function (solid line) and standard error of measurement (dashed line) for the AMT. Left-hand graph: younger group; Right-hand graph: older group. Specificity and SEM are measured on a standardized scale. Probability = the precision of the test at a particular point, measured in a squared metric. SEM = the precision with which an individual’s level of ability on the AMT can be estimated using the current scale. The SEM is lowest, and hence estimates are more precise, where the test information function is at its peak.

Fig 1 shows the test information function. Fig 1 shows that the point where the information reaches its maximum level is less than zero in the latent trait of memory specificity in both groups. The standard error of measurement corresponding to a score of 0 in the latent trait is 0.28 in both younger and older groups. The ranges of the latent trait where the standard error of measurement is lower than 0.5 varies between -4.6 and 0.6 and between -5.6 and 1.2 in young and older groups, respectively. Outside this range, the SEM increases rapidly in higher scores on specificity.

### Differential item functioning

The results of the DIF analysis for the AMT items are summarized in Table 4. Three items exhibited significant DIF: *Happiness*, *Energy and Guilty*. These items showed a total DIF effect: scores in these items affected overall AMT scores. Additionally, *Guilty* and *Happiness* also showed uniform DIF: younger participants found it easier to retrieve a specific memory in response to *Guilty* and more difficult to retrieve a specific memory in response to *Happiness* than older participants. *Happiness* also shows a non-uniform DIF: in the older group, this item shows a bad discrimination (a low item-slope) between low and high scorers in the specificity latent trait. Nevertheless, its discrimination in the young group is good (item-slope higher than .30).

**Table 4:** Effect of the amount of uniform, non-uniform, and overall DIF present by Item.

	McFadden’s *R*^2^Δ		
Item	Uniform DIF	Non-Uniform DIF	Overall DIF
Happiness	.059*	.038*	.097**
Friendship	.005	< .001	.005
Excitement	.003	< .001	.004
Energy	.019	.019	.038*
Smile	.002	.001	.002
Guilty	.052*	.003	.055*
Failure	.004	< .001	.004
Worry	< .001	< .001	< .001
Sadness	< .001	< .001	.001
Illness	< .001	.001	.001

Note.

*: medium effect size

**: large effect size.

**Fig 2:**
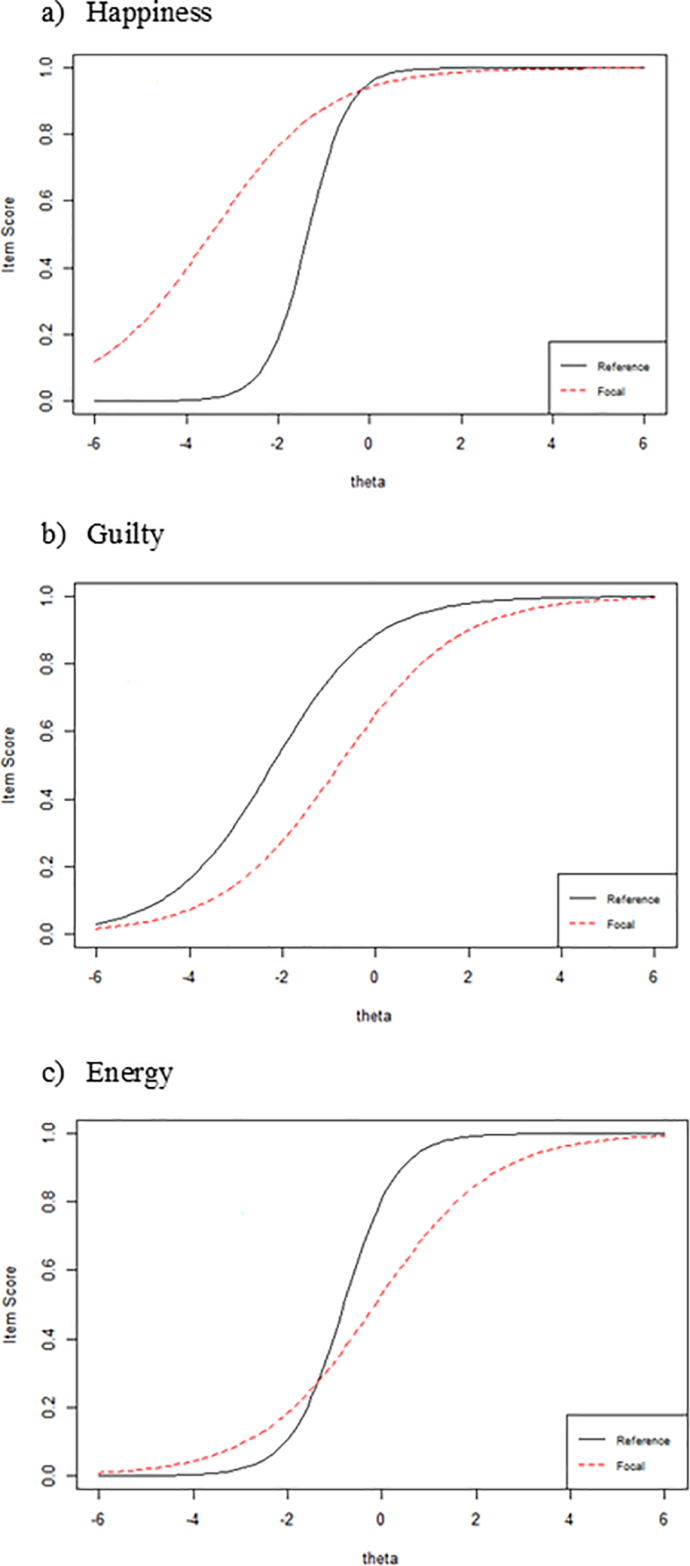
Item response functions for DIF items between young and older groups. Reference: young group; Focal: older group. For an item exhibiting no age group-DIF, the red and black lines would coincide so that both groups would have the same probability (item score) of responding with a specific memory at every point along the latent trait (theta). Uniform and non-uniform DIF manifest themselves as parallel and non-parallel item response functions, respectively.

Fig 2 shows the item response functions for DIF items.

**Fig 3:**
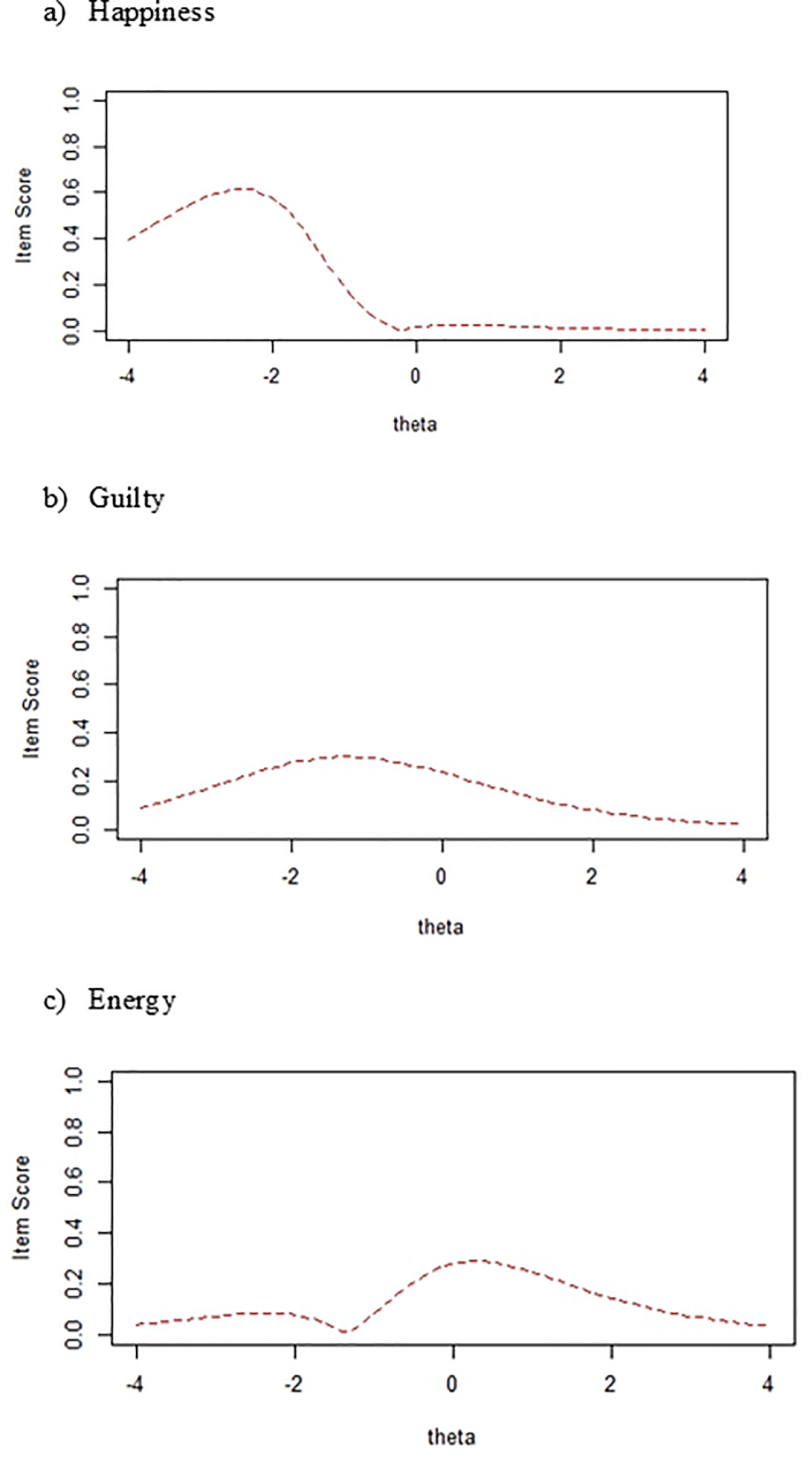
Differences in true scores for DIF items between young and older groups. Items scores are presented as standardized. The line shows the absolute difference between the Item Characteristic Curves for the two groups (young and older). Where the line is higher indicates at what level of the latent trait Specificity (theta) the difference is greater.

Fig 3 shows differences in item true scores (presented as standardized) between young and older groups in the AMT items flagged for DIF. Fig 3 shows that the greatest differences between both groups (young and older) appear in scores below the mean in the specificity latent trait in *Happiness* and *Guilty*. Nevertheless, as regards the cue word *Energy*, the greatest differences between groups appear in scores around the mean.

## Discussion

In view of the lack of studies validating the AMT in Spanish populations and, specifically in older groups, this work aims to validate and analyze the psychometric properties of a Spanish version of the AMT in two groups of different ages (young and older adults). To this end, first, we analyzed the factor structure of both tests, and using an IRT analysis, we assessed the psychometric properties of the AMT to determine their applicability in both populations. Second, we also analyzed the DIF of the AMT cue words to determine whether any of the AMT cue words are differentially related to OGM according to age group.

Regarding the factor structure of the AMT, the results for both samples (young and older) show a one-factor structure comprising positive and negative cue word items. This finding coincides with previous studies in different populations [13,14,16], suggesting the AMT measures the unidimensional construct of AM specificity.

With regard to the psychometric properties of the AMT, the IRT analyses show that, broadly speaking, all items present good discrimination indices in both age groups (except the *Happiness* item in the older group). However, the item thresholds show that the thresholds for retrieval of specific memories are generally lower than the mean in both the young and older adults groups, thus suggesting the task is easy for both age groups. Finally, regarding the precision of the test, the measures obtained are more exact (i.e., measurement errors are the lowest) in both young and older individuals scoring below and around the mean of the memory specificity latent trait. Measures obtained in individuals scoring above the mean of the memory specificity latent trait are less accurate (i.e., measurement errors are higher) so that, as the individual’s score in the memory specificity factor increases, the measurement error also does. Nevertheless, it is worth noting that the SEM was equal or below 1.0 for the range of AM specificity studied, which suggests that the AMT is a precise test in both young and older samples.

Our results show that the young group retrieved more specific memories than the older group. This result is consistent with studies that suggest aging is associated with a reduction in AM specificity [17,29]. To ensure that our results can be interpreted as evidence of lower levels of OGM trait in young adults compared to older adults, a DIF analysis was performed. This analysis found evidence of difference by age group in the functioning of three AMT items: *Happiness*, *Guilty* and *Energy* scores affect the overall score obtained on the AMT. Regarding the cue word *Happiness*, results suggest that, in participants with lower levels of specificity, older adults are more likely to respond with a specific memory than young adults. This greater specificity might be associated with the age-related bias for positivity in memory [52]. This positivity effect could facilitate the recall of happy events even in individuals with low scores on the specificity trait.

As regards the cue word *Guilty*, results show that older adults are less likely to recall specific events than young individuals, mainly when they score lower than average on AM specificity. Considering that older adults tend to be less specific, the difference between young and older adults in the likelihood of responding to this cue word with a specific memory might be expected to increase among participants who already exhibit low memory specificity.

As regards the cue word *Energy*, we find that the likelihood of individuals with a low score on the specificity trait generating a specific memory is close to zero (in both older and young adults). Individuals scoring close to, or higher than the mean, on the specificity trait are more likely to retrieve a specific memory and this is where the differences between the young and the older group emerge. Analyzing the item thresholds, we find that *Energy* is the most difficult item for both groups. Investigations have shown that participants retrieve more specific memories in response to cues high in imageability (e.g., “car”) than in response to low imageability cues (e.g., “wisdom”) [53]. As can be seen in Table 1, e*nergy* can be considered a low imageability cue word. This would explain why the likelihood of individuals with low specificity levels retrieving a specific memory in response to this cue word is low. Similarly, in those with medium and high specificity levels, the likelihood of recalling a specific memory increases, although this increase is less notable in older adults. Thus, if older adults are generally less specific than young adults, the use of low imageability cue words, which are therefore more difficult, could accentuate this age-related difference.

This work has a series of limitations. First, as indicated by Griffith et al. [1], there is currently no generally accepted standardized set of cue words, hence the idiosyncrasies of each particular cue could have an impact on the results obtained on the AMT. Second, past history of psychological disorders of our participants has not been assessed. Different studies suggest that autobiographical memory remains overgeneral in individuals with a history of emotional disorder, even if they are not currently in an episode [54–56]. Although our participants did not show any psychiatric disorders at the moment of the assessment, previous history of psychiatric disorders could be influencing AMT performance. Finally, another limitation is that both groups (older and younger) differ in educational level and the presence of differences in IQ scores between groups has not been tested. Differences in IQ levels or educational level might account for overgeneral memory, and not all studies have taken adequate account of this possibility. Some studies that have controlled for number of years in education or IQ [9, 57–61] have found that differences remain even after matching for such variables. Nevertheless, other studies have found that IQ is a significant predictor of overgeneral memory [14,62]. Given these different findings, it is evident that the effect of educational and IQ variables in overgeneral memory still needs to be clarified and future research should take this into account.

In summary, the Spanish version of the AMT seems to be relatively easy for both study samples. Indeed, Griffith et al. suggest that the AMT may be too easy for individuals with a high level of cognitive function [13]. Although normal aging is often accompanied by a deterioration of different cognitive skills and, as expected, older adults remembered in a more overgeneral way than did young people in our study, our older group also found the AMT easy to perform. As our older group comprises participants that routinely perform intellectual activities, our results cannot be generalized to lower functioning older adults. Future research should analyze the functioning of the AMT in non-clinical populations with varying levels of cognitive function. Our study also shows that three of the items in the AMT function differently depending on age, and hence their use is inappropriate when comparing memory specificity across age groups. Consequently, we consider it important to continue research on the validation of the AMT in order to establish a standardized set of cue words adapted to young and older populations. For example, the DIF may be caused by the meaning and significance of certain words changing over time. In a longitudinal study, asking people to recall their childhood at different points in their lives, Field [63] found that autobiographical memories became increasingly positive with age. This fact is congruent with Socioemotional Selectivity Theory [64] which posits that the age-associated motivational shifts towards emotional goals and, as a consequence, the emotional information recalled by older people is disproportionately positive compared to young people. It may be that the autobiographical memories retrieved in response to the cue words change their characteristics as people get older. For example, McAdams [65] found that individuals who have experienced difficult life events tend to narrate their events with a positive evaluation (“redemption effect”). The redemption effect can be defined as an explicit transformation of an autobiographical event that is decidedly negative to a decidedly positive affect. Could be this effect more common as we get older? Longitudinal studies of AMT performance could help to clarify whether this positivity effect is to some extent responsible for the differences between young and older adults in AMT performance. In any event, and given the results of this study, we consider it unadvisable to use *Happiness*, *Guilty* and *Energy* as cue words in future studies that compare AMT performance between young and older adults. Finally, it might be considered appropriate to include the rest of the cue words used in this AMT version as part of a standardized set of cue words of the AMT for young and older populations.
